# Exposure to ambient benzene, genetic susceptibility, and incident rheumatic diseases in the UK

**DOI:** 10.3389/fpubh.2026.1931281

**Published:** 2026-10-08

**Authors:** Yangchang Zhang, Dongni Huang, Yang Pu, Bin Wu

**Affiliations:** 1 Department of Rheumatology and Immunology, The First Affiliated Hospital of Chongqing University of Chinese Medicine, Chongqing, China; 2 Department of Obstetrics and Gynecology, Women and Children's Hospital of Chongqing Medical University (Chongqing Health Center for Women and Children), Chongqing, China; 3 School of General Education, Chongqing College of Traditional Chinese Medicine, Chongqing, China

**Keywords:** ambient benzene levels, genetic susceptibility, gout, rheumatoid arthritis, systemic lupus erythematosus

## Abstract

**Background:**

Systemic lupus erythematosus (SLE), rheumatoid arthritis (RA), and gout are prevalent rheumatic diseases.

**Methods:**

We examined the associations of long-term ambient benzene exposure and genetic susceptibility with incident SLE, RA, and gout, as well as their joint associations with disease risk.

**Results:**

Among participants with available ambient benzene estimates during the median follow-up of 13.6 to 13.7 years, 469 SLE, 7,494 RA, and 9,816 gout cases occurred during follow-up. Compared with participants with the lowest benzene exposure, those with the highest exposure had approximately 39, 17, and 26% higher risks of SLE, RA, and gout, respectively. Participants with both high benzene exposure and high genetic susceptibility consistently showed the highest disease risks.

**Conclusion:**

This large UK cohort study found that both ambient benzene exposure and genetic risk factors were collectively associated with increased risks of SLE, RA, and gout.

## 1 Introduction

Rheumatic diseases (RDs) constitute a major public health threat by contributing disproportionately to chronic morbidity, disability, reduced quality of life, and escalating health care expenditures globally (1–5). There is a close association between RDs, such as RA and a wide array of comorbidities, such as cardiovascular disease, interstitial lung disease, osteoporosis and fractures, infections, and renal impairment, along with an increased risk of dying prematurely (6–10). Current therapies can alleviate clinical symptoms and delay disease progression, yet most patients require continuous monitoring and long-term maintenance treatment, leading to considerable health care resource utilization and substantial economic burden. Therefore, early prevention and intervention for these diseases are of critical importance for reducing the risk of disease progression, ameliorating inflammation, and improving quality of life (11). Since the early 21st century, the incidence and prevalence of RDs have continued to rise or remain persistently high in many regions, with the burden particularly pronounced in high-income regions and increasing rapidly in low- and middle-resource settings, thereby posing ongoing challenges to public health (3, 4, 12). Previous evidence suggests that genetic susceptibility and environmental exposures may jointly contribute to the development of SLE (13). Exploring their interaction is critical for elucidating disease mechanisms and advancing the identification and precise prevention of high-risk populations.

Recognized for its hematotoxic and cancer-causing properties, benzene is a volatile organic compound prevalent in urban and industrial settings, posing a major environmental pollution threat (14–17). Benzene exposure occurs primarily through inhalation of ambient and indoor air, with common sources including vehicle exhaust, petrol evaporation, tobacco smoke, domestic fuel combustion, and industrial emissions.

Prolonged low-level exposure to benzene occurs primarily through inhalation of contaminated air, particularly in settings with high traffic density, significant industrial emissions, or indoor tobacco smoke. Although the adverse effects of benzene on the hematopoietic and immune systems are well documented, including hematotoxicity, leukemogenesis, and immunological dysfunction (18–20), its potential role in the pathogenesis of RDs remains incompletely understood. Emerging evidence indicates that benzene exposure may induce oxidative stress and dysregulation of inflammatory pathways (18, 21, 22). However, epidemiological studies evaluating the impact of ambient benzene exposure on the risk of RDs are limited, and the possibility of gene–environment interactions has yet to be systematically explored.

Therefore, we sought to conduct a prospective investigation of the association between long-term ambient benzene exposure and the incidence of major RDs, including RA, SLE and gout. This investigation draws on data from the UK Biobank, a large-scale cohort with approximately 500,000 member (23). Drawing on the high-resolution genetic data available in the UK Biobank, we derived disease-specific polygenic risk scores that integrate numerous genetic loci, allowing for comprehensive assessment of individual genetic predisposition (24, 25). This design enabled us to systematically evaluate not only the independent effects of ambient benzene exposure but also the potential gene–environment interactions between polygenic risk and ambient benzene exposure on the risk of incident RDs.

## 2 Materials and methods

### 2.1 Study population

Our study sourced data from the UK Biobank, a nationwide longitudinal study recruited 0.5 million populations in UK during 2006 and 2010 (23). Most participants were about 40 years or older. The participants remained under observation during the study period, unless they withdrew. An array of data information, including demographics, examinations, medical conditions, biological specimens, and imaging were collected methodically through touchscreen questionnaire, interviews, and clinical records (23). The North West Multi-Center Research Ethics Committee granted ethics approval to the UK Biobank for research involving populations (No.21/NW/0157). All participants signed a form indicating their informed consent. Individuals with less than one year of follow-up, a prior RDs diagnosis, missing ambient benzene exposure data, or unavailable genetic data were excluded from the study. Finally, subsamples of 483,064, 480,810 and 483,130 participants were included in the analyses of SLE, RA and gout with the exposure to ambient benzene, respectively (Supplementary Figure S1). The median follow-up duration was 13.7 years for SLE, 13.6 years for RA, and 13.6 years for gout.

### 2.2 Air pollution exposure assessment

The UK Department for Environment, Food and Rural Affairs (DEFRA) provided annual mean ambient benzene concentration estimates (μg/m^3^) at a spatial resolution of 1 × 1 km. These estimates were derived from the Pollution Climate Mapping (PCM) framework, which provides modeled background concentrations of air pollutants across the UK and has been widely used in environmental health research (26–28). The PCM framework integrates national emissions data and pollutant-specific modeling procedures to estimate spatial variations in ambient pollutant concentrations. Further details regarding the modeling framework and its application are available in DEFRA technical reports (29). Geospatial information for each participant was obtained from the UK Biobank, and the modeled benzene concentrations were linked to participants’ residential locations. Annual ambient benzene concentrations were treated as time-varying exposures in the primary analyses, with exposure values updated according to calendar year and residential address information. These concentrations represent modeled outdoor background benzene levels at participants’ residential locations and are consistent with low-level environmental exposure in the general population rather than occupational or acute high-dose exposure. The tertiles of mean concentrations of benzene exposure were 0.153–0.571 μg/m^3^ for low exposure, 0.572–0.741 μg/m^3^ for moderate exposure, and 0.741–2.620 μg/m^3^ for high exposure in the UK from 2003 to 2010 (Supplementary Figure S2).

### 2.3 Polygenic risk score

PRS datasets for RA (Field ID: 26273) and SLE (Field ID: 26278) were obtained from the UK Biobank Research Access Platform. These standardized scores were derived exclusively from external genome-wide association studies (GWAS) using trait-specific Bayesian meta-analyses that integrated data across ancestries and related traits to enhance predictive accuracy. For each participant, PRS was calculated as the genome-wide sum of allele dosages weighted by posterior effect estimates, following uniform disease and trait definitions to ensure methodological consistency. Detailed procedures and technical methods have been reported in the previous study (25). Details of the single-nucleotide polymorphism (SNP) genotyping process, imputation, and quality control have been described previously (30). Gout PRS were generated using the imputed genetic data from the UK Biobank and GWAS summary statistics from the FinnGen consortium (M13_Gout, release R12) (31), which included 12,342 cases and 315,115 controls of European ancestry. Then, a total of 22 independent SNPs (*r*^2^ < 0.001 and 10,000 kb clumping window) with *p* < 5 × 10^−8^ were selected (Supplementary Table S6). Individual PRS for gout were computed using PLINK v1.9 as the weighted sum of risk alleles across these loci, using GWAS effect sizes as weights (32). Participants were subsequently stratified into low (tertile 1), intermediate (tertile 2), and high (tertile 3) genetic risk groups according to their PRS distribution.

### 2.4 Outcome ascertainment

Incident cases of RA, SLE and gout were identified through linked hospital admissions using International Classification of Diseases, 10th Revision (ICD-10) codes in the UK Biobank. ICD-10 definitions for outcomes were provided in Supplementary Table S1. Dates of death were obtained from the Office for National Statistics mortality register. Follow-up spanned baseline to the earliest disease onset, death or loss to follow up. The censoring date was October 31, 2022.

### 2.5 Covariates

A series of covariates were selected into our analyses, including age (< 65 years and ≥ 65 years), Sex (Male and Female), ethnicity (White and Non-white), educational levels (College and Other levels), Body mass index (BMI) (kg/m^2^) (Underweight, Normal, Overweight, and Obese), alcohol drinking (Current, Previous and Never), smoking status (Current, Previous and Never), household income (< £ 31,000 and ≥ £ 31,000), average outdoor time (hours) and diet score (ranged from 0–7).

### 2.6 Statistical analyses

Baseline characteristics across quartiles of benzene exposure were summarized as means (SD) for continuous variables and counts (percentages) for categorical variables, with group differences tested using analysis of variance or χ^2^ tests as appropriate. Missing covariates were treated as an independent indicator. We employed time-varying Cox proportional hazards models to examine the associations of ambient benzene exposure and PRS with RA, SLE and gout. Schoenfeld residuals were used to verify the proportional hazards assumption. The associations between ambient benzene exposure and RDs were described as hazard ratios (HRs) and the corresponding 95% confidence intervals (95% CIs). Ambient benzene exposure was treated as continuous or quartiles. The E-values show the least amount of association an unmeasured confounder would need with both the exposure and the outcome to completely explain the observed association (33). A restricted cubic spline (RCS) model using three knots was applied to examine the dose–response associations between ambient benzene exposure and RDs. The numbers of knots were determined using minimum Akaike Information Criterion (AIC).

To assess the effects of genetic risk on the association between ambient benzene exposure and RDs, we conducted a subgroup analysis stratified by PRS tertiles and calculated multiplicative interaction. To assess interaction on the additive scale, we calculated the relative excess risk due to interaction (RERI), attributable proportion due to interaction (AP), and synergy index (SI), together with their 95% CIs. RERI quantifies excess risk beyond individual effects, and AP represents the proportion of risk attributable to the interaction. We evaluated the joint effects of ambient benzene exposure and PRS on incident rheumatoid arthritis, systemic lupus erythematosus and gout, using participants with low genetic risk and low ambient benzene exposure as the reference group.

We conducted a series of sensitivity analyses to test the robustness of our findings. We repeated the main models after restricting the sample to participants who had lived at their baseline address for at least 5 years, excluding those reporting poor self-rated health at baseline, and restricting analyses to white participants.

All analyses were performed in R (version 4.4.1), and statistical significance was defined as two-sided *p* < 0.05.

## 3 Results

Baseline characteristics of participants across quartiles of benzene exposure were presented in Table 1. Among the population with available ambient benzene exposure at baseline, the quartiles of ambient benzene exposure ranged from 0.15 to 0.40 μg/m^3^ for Q1, 0.40 to 0.47 μg/m^3^ for Q2, 0.47 to 0.56 μg/m^3^ for Q3, and 0.56 to 1.73 μg/m^3^ for Q4. Baseline demographic, socioeconomic, and lifestyle characteristics differed across quartiles of ambient benzene exposure (Table 1).

**Table 1:** Baseline characteristics of included participants.

Ambient benzene exposure (μg/m^3^)					
Variables	Q1 (0.15 ~ 0.40]	Q2 (0.40 ~ 0.47]	Q3 (0.47 ~ 0.56]	Q4 (0.56 ~ 1.73]	*p*
	*n* = 124,983	*n* = 125,022	*n* = 124,929	*n* = 124,966	
Age, *n* (%)					
< 65	99,295 (79.45)	100,503 (80.39)	101,338 (81.12)	103,101 (82.50)	<0.001
> = 65	25,688 (20.55)	24,519 (19.61)	23,591 (18.88)	21,865 (17.50)	
Sex, *n* (%)					
Female	68,554 (54.85)	67,979 (54.37)	67,831 (54.30)	67,630 (54.12)	0.002
Male	56,429 (45.15)	57,043 (45.63)	57,098 (45.70)	57,336 (45.88)	
Ethnicity, *n* (%)					
White	122,769 (98.23)	121,529 (97.21)	118,816 (95.11)	107,262 (85.83)	<0.001
Non-White	1894 (1.52)	3,149 (2.52)	5,683 (4.55)	16,936 (13.55)	
Missing	320 (0.26)	344 (0.28)	430 (0.34)	768 (0.61)	
Education, *n* (%)					
College	43,187 (34.55)	37,614 (30.09)	35,011 (28.02)	44,298 (35.45)	<0.001
Other levels	61,553 (49.25)	64,172 (51.33)	64,933 (51.98)	58,809 (47.06)	
Unknown	20,243 (16.20)	23,236 (18.59)	24,985 (20.00)	21,859 (17.49)	
BMI, *n* (%)					
Obese	28,317 (22.66)	30,388 (24.31)	32,201 (25.78)	30,744 (24.60)	<0.001
Overweight	53,875 (43.11)	53,755 (43.00)	52,767 (42.24)	50,677 (40.55)	
Normal	41,710 (33.37)	39,698 (31.75)	38,605 (30.90)	41,467 (33.18)	
Underweight	609 (0.49)	606 (0.48)	593 (0.47)	799 (0.64)	
Missing	472 (0.38)	575 (0.46)	763 (0.61)	1,279 (1.02)	
Drinking status, *n* (%)					
Current	116,743 (93.41)	115,753 (92.59)	114,618 (91.75)	110,960 (88.79)	<0.001
Previous	3,813 (3.05)	4,317 (3.45)	4,548 (3.64)	5,286 (4.23)	
Never	4,210 (3.37)	4,726 (3.78)	5,394 (4.32)	7,894 (6.32)	
Missing	217 (0.17)	226 (0.18)	369 (0.30)	826 (0.66)	
Smoking status, *n* (%)					
Current	10,744 (8.60)	12,333 (9.86)	13,637 (10.92)	15,838 (12.67)	<0.001
Previous	42,836 (34.27)	43,150 (34.51)	43,370 (34.72)	42,803 (34.25)	
Never	70,903 (56.73)	69,003 (55.19)	67,202 (53.79)	65,155 (52.14)	
Missing	500 (0.40)	536 (0.43)	720 (0.58)	1,170 (0.94)	
Household income (£), *n* (%)					
< 31,000	47,812 (38.25)	52,516 (42.01)	54,519 (43.64)	49,414 (39.54)	<0.001
> = 31,000	59,868 (47.90)	54,443 (43.55)	51,462 (41.19)	53,145 (42.53)	
Missing	17,303 (13.84)	18,063 (14.45)	18,948 (15.17)	22,407 (17.93)	
Outdoor time (hours), *n* (%)					
(0–2]	34,784 (27.83)	35,293 (28.23)	35,159 (28.14)	37,105 (29.69)	<0.001
(2–3.5]	33,240 (26.60)	32,644 (26.11)	31,745 (25.41)	29,818 (23.86)	
(3.5–24]	29,994 (24.00)	29,164 (23.33)	28,852 (23.09)	26,110 (20.89)	
Missing	26,965 (21.57)	27,921 (22.33)	29,173 (23.35)	31,933 (25.55)	
Diet score, *n* (%)					
0–1	18,641 (14.91)	20,062 (16.05)	21,011 (16.82)	18,581 (14.87)	<0.001
2–3	69,385 (55.52)	68,561 (54.84)	68,332 (54.70)	67,774 (54.23)	
4–5	35,070 (28.06)	34,511 (27.60)	33,552 (26.86)	35,853 (28.69)	
6–7	1775 (1.42)	1779 (1.42)	1817 (1.45)	2,165 (1.73)	
Missing	112 (0.09)	109 (0.09)	217 (0.17)	593 (0.47)	

During the median follow-up time of over 13 years, a total of 469 incident cases of SLE, 7,494 cases of RA, and 9,816 cases of gout were documented (Table 2). An SD increased ambient benzene levels were associated with a higher risk of SLE (HR:1.11, 95% CI:1.02, 1.21), RA (HR:1.04, 95% CI:1.02, 1.07), and gout (HR: 1.10, 95% CI: 1.07, 1.12). The corresponding E-values were 1.46, 1.24 and 1.43. The HRs and 95% CIs of the highest ambient benzene levels (Q4) were 1.39 (95% CIs: 1.06, 1.84) for SLE, 1.17 (95% CIs:1.09, 1.25) for RA and 1.26 (95% CIs: 1.19, 1.34) for gout, compared to the lowest quartile (Q1). The observed E-values ranged from 1.62 to 2.12, indicating that only unmeasured confounders of considerable strength could fully account for these associations.

**Table 2:** Associations of ambient benzene exposure with risk of rheumatic diseases.

Benzene exposure (μg/m^3^)	Cases/Person-years (Incident rate per 100,000 person-years)	HR (95% CI)	*p* value	*P* for trend	E-value
Systemic lupus erythematosus, SLE				0.022	
Q1, lowest	32/588327 (5.44)	1 [reference]	NA		NA
Q2	93/1615713 (5.76)	1.23 (0.94–1.62)	0.133		1.76
Q3	171/2199120 (7.78)	1.29 (0.98–1.71)	0.074		1.90
Q4, highest	173/2031035 (8.52)	1.39 (1.06–1.84)	0.019		2.12
Per SD increases	469/6434194 (7.29)	1.11 (1.02, 1.21)	0.012	NA	1.46
Rheumatoid Arthritis, RA				< 0.001	
Q1, lowest	500/585156 (85.45)	1 [reference]	NA		NA
Q2	1752/1602015 (109.36)	1.19 (1.12–1.28)	< 0.001		1.67
Q3	2730/2171262 (125.73)	1.18 (1.10–1.26)	< 0.001		1.64
Q4, highest	2512/2008734 (125.05)	1.17 (1.09–1.25)	< 0.001		1.62
Per SD increases	7494/6367167 (117.70)	1.04 (1.02, 1.07)	< 0.001	NA	1.24
Gout					
Q1, lowest	575/588448 (97.71)	1 [reference]		< 0.001	NA
Q2	2351/1608928 (146.12)	1.15 (1.08–1.21)	< 0.001		1.57
Q3	3536/2180604 (162.16)	1.29 (1.22–1.37)	< 0.001		1.90
Q4, highest	3354/2016529 (166.33)	1.26 (1.19–1.34)	< 0.001		1.83
Per SD increases	9816/6394509 (153.51)	1.10 (1.07, 1.12)	< 0.001	NA	1.43

Model adjusted for sex, age, ethnicity, household income, education, body mass index (BMI), drinking status, smoking status, outdoor time, and diet score.

The dose-associations of ambient benzene exposure and RDs were presented in Figure 1. Non-linear trends were found in RA (*P* for nonlinearity < 0.001) and gout (*P* for nonlinearity < 0.001). Their overall trend showed a rapid increase followed by a gradual decrease. Then, a monotonic increasing trend was observed in the risk of SLE (*P* for nonlinearity = 0.281).

**Figure 1:**
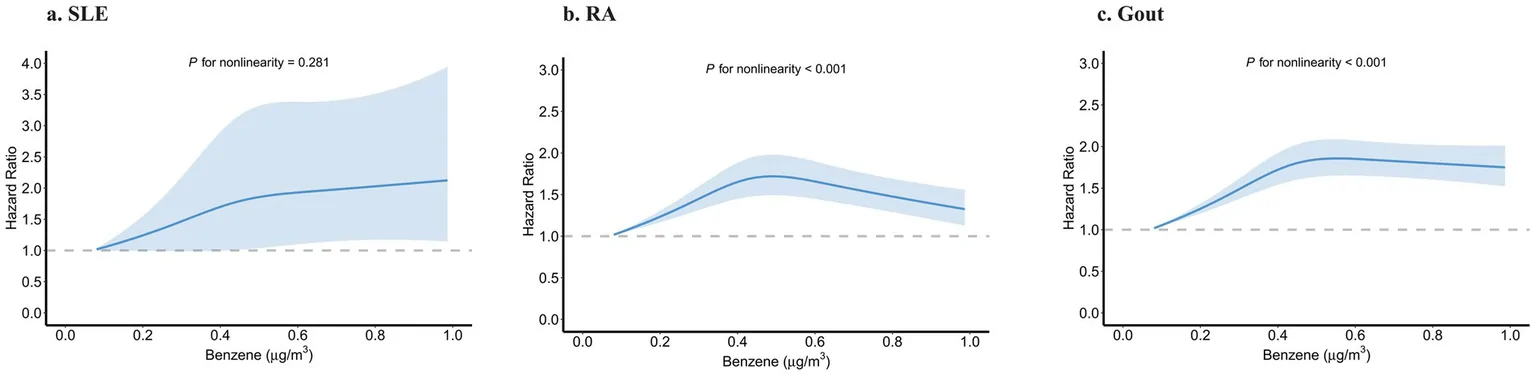
Associations of long-term exposure to benzene with the risk of rheumatic diseases. A restricted cubic spline regression model with 3 knots was used to estimate the dose–response associations between ambient benzene exposure and the risk of rheumatic diseases. HRs and 95% CIs were adjusted for sex, age, ethnicity, household income, education, body mass index (BMI), drinking status, smoking status, outdoor time, and diet score.

In the stratified analysis by PRS, we did not observe any multiplicative interaction between ambient benzene exposure and PRS on SLE (*P* for interaction = 0.41), RA (*P* for interaction = 0.46) and gout risk (*P* for interaction = 0.26) (Table 3). Additionally, the joint associations of ambient benzene exposure and PRS with RDs risk were examined (Figure 2). Higher levels of benzene exposure and genetic risk were jointly associated with a higher incidence of RA, SLE and gout, with evidence of a linear trend (*P* for trend < 0.001). We observed the strongest associations with the highest levels of ambient benzene exposure and genetic risk (SLE: HR: 4.15; 95% CI: 2.84, 6.08; RA: HR: 2.02; 95% CI:1.86, 2.20; gout: HR: 2.76; 95% CI:2.55, 2.98), compared with those with low levels of both exposure and genetic risk. We cannot find an additive scale interaction between PRS and ambient benzene exposure on the risk of incident SLE, RA and gout in the UK participants (The RERI and AP estimates crossed 0) (Supplementary Table S2).

**Table 3:** Associations of ambient benzene exposure with risk of rheumatic diseases stratified by the genetic risk group.

Incidence	Ambient benzene exposure (μg/m^3^)		*P* for interaction
	Low levels (< 0.43)	High levels (≥ 0.43)	
Systemic lupus erythematosus, SLE			
Low genetic risk			0.41
Cases/person-years	23/717497	60/1427090	
HR (95% CI)	1 [reference]	1.34 (0.84–2.16)	
Intermediate genetic risk			
Cases/person-years	32/734406	79/1410459	
HR (95% CI)	1 [reference]	1.31 (0.89–1.93)	
High genetic risk			
Cases/person-years	70/752137	205/1392606	
HR (95% CI)	1 [reference]	1.13 (0.87–1.46)	
Rheumatoid Arthritis, RA			
Low genetic risk			0.46
Cases/person-years	520/723565	1255/1399047	
HR (95% CI)	1 [reference]	1.06 (0.96–1.17)	
Intermediate genetic risk			
Cases/person-years	691/731764	1638/1390601	
HR (95% CI)	1 [reference]	1.11 (1.01–1.21)	
High genetic risk			
Cases/person-years	1041/731842	2349/1390349	
HR (95% CI)	1 [reference]	1.04 (0.97–1.12)	
Gout			
Low genetic risk			0.26
Cases/person-years	547/708633	1510/1423068	
HR (95% CI)	1 [reference]	1.24 (1.13–1.37)	
Intermediate genetic risk			
Cases/person-years	977/737611	2251/1393984	
HR (95% CI)	1 [reference]	1.22 (1.13–1.31)	
High genetic risk			
Cases/person-years	1402/751132	3129/1380081	
HR (95% CI)	1 [reference]	1.16 (1.09–1.23)	

Model adjusted for sex, age, ethnicity, household income, education, body mass index (BMI), drinking status, smoking status, outdoor time, and diet score. Ambient benzene exposure was dichotomized at the median (< 0.43 vs ≥ 0.43 μg/m^3^).

**Figure 2:**
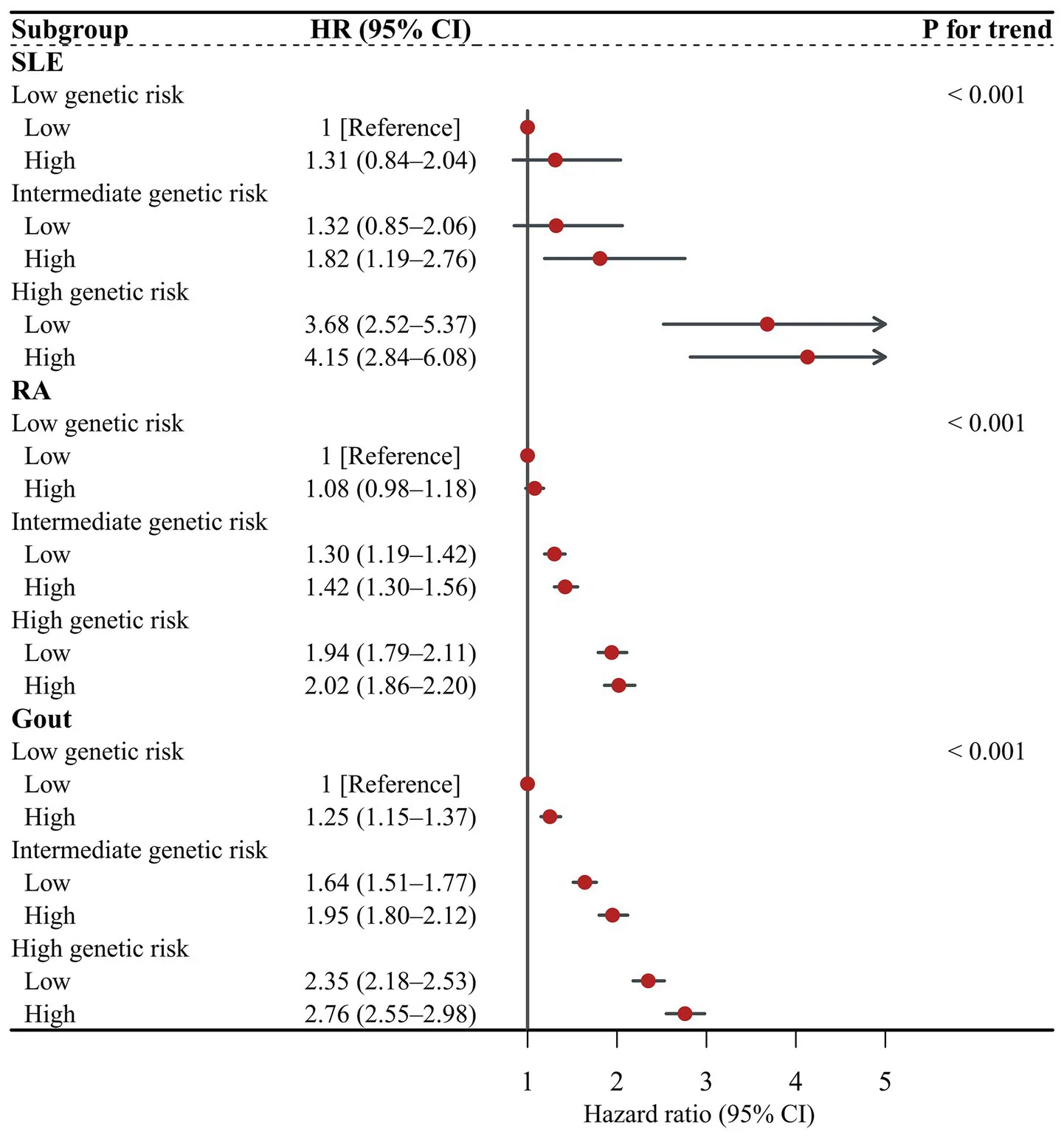
Joint associations of long-term ambient benzene exposure and genetic risk with the risk of incident rheumatic diseases. Model adjusted for sex, age, ethnicity, household income, education, body mass index (BMI), drinking status, smoking status, outdoor time, and diet score. These analyses represent joint associations across categories of benzene exposure and genetic risk rather than statistical interactions.

Our main results did not materially differ across certain sensitivity analyses. Consistent associations were observed after multiple sensitivity analyses, including restricting to participants who had lived at their current location for more than 5 years (Supplementary Table S3), excluding those with poor self-reported health (Supplementary Table S4), and restricting to white participants (Supplementary Table S5).

## 4 Discussion

This large-scale prospective cohort of nearly 500,000 adults in the UK found that increased ambient benzene exposure was consistently associated with increased risks of SLE, RA, and gout. Joint analyses showed that participants with both high benzene exposure and elevated genetic risk consistently had the highest observed risks of SLE, RA, and gout. Our study suggests that ambient benzene exposure may be associated with an increased risk of autoimmune and inflammatory diseases.

Earlier occupational studies have established that benzene exposure can induce hematotoxicity (19), immune suppression (18), and leukemogenesis (20), but translation to autoimmune disease risk has remained uncertain. Recent epidemiological evidence further supports associations between air pollution and RDs. Long-term exposure to particulate air pollution has been associated with an increased risk of immune-mediated diseases (13, 34). Two cohort studies have reported also the associations of ambient air pollutants with incident RA and SLE, particularly among individuals with high genetic susceptibility (13, 35). These findings provide broader epidemiological context for the observed association between ambient benzene exposure and RDs risk. Nevertheless, epidemiological evidence specifically linking ambient benzene exposure to RDs remains limited. Our findings suggest that ambient benzene exposure may be associated with an increased risk of RDs. Previous reviews also indicated that exposure to BTEX can cause human respiratory, cardiovascular, blood, reproductive, and neural functions disorders (36). These findings are consistent with emerging evidence suggesting that low-level benzene exposure may exert clinically relevant effects (37, 38).

The present findings underscore the regulatory importance of low-level benzene exposures. Elevated risks of RA, SLE, and gout were observed across exposure ranges commonly encountered in daily environments, raising concerns about potential health effects of low-level environmental exposure. Although the dose–response curves varied across diseases, with a more linear pattern for SLE and plateauing tendencies for RA, and gout, the consistent observation of elevated risks suggests that benzene exposure may be associated with disease risk across a broad exposure spectrum. These results suggest that existing standards, which are largely based on carcinogenic and hematotoxic outcomes, may underestimate the broader health implications of benzene. By delineating disease-specific exposure–response profiles, this study provides an empirical basis for refining risk thresholds and supports the integration of autoimmune and inflammatory outcomes into future air quality guidelines. Furthermore, this evidence reinforces the importance of prioritizing emission reduction strategies targeting traffic, industrial, and indoor sources, which may yield public health benefits extending beyond cancer prevention.

Several biological mechanisms may underlie the observed associations based on the existed research. Benzene metabolites, including hydroquinone and benzoquinone, can induce oxidative stress and DNA damage in hematopoietic and immune cells (22, 39, 40). Such cellular perturbations may contribute to immune dysfunction and altered cellular metabolism. In SLE, macrophages undergo a glycolytic shift regulated by HIF-1α and mTOR signaling, which amplifies proinflammatory mediator production and sustains chronic activation (41). In RA, enhanced glycolytic metabolism contributes to the proinflammatory phenotype of macrophages and fibroblast-like synoviocytes, whereas blockade of glycolysis attenuates inflammatory responses in both cell types (42). In gout, monosodium urate crystals activate the NLRP3 inflammasome, promoting the maturation and release of IL-1β and triggering an inflammatory response (43). Recent mechanistic evidence further supports the potential role of air pollution in immune dysregulation underlying RDs. Zhao et al. reported that PM_10_ exposure may impair CD56^dim^ NK-cell cytotoxicity through FNBP1 suppression, thereby weakening immune surveillance and exacerbating inflammatory responses in RA (44). Although these findings were derived from particulate matter rather than benzene exposure, they provide additional biological plausibility for the involvement of air pollution–related immune dysregulation in RDs development. Collectively, these mechanisms provide biologically plausible explanations for the observed associations.

Beyond the direct metabolic and immunotoxic effects of benzene, genetic predisposition may influence individual vulnerability to these exposures. In our study, PRS were derived from genome-wide significant variants identified in large-scale GWAS, capturing overall inherited susceptibility. Several loci included in the score have well-characterized functional roles. ABCG2 rs2231142 (Q141K) impairs urate transport and is a well-established risk variant for gout (45); GCKR rs1260326 (P446L) is a functional variant involved in the regulation of glucose and lipid metabolism (46). Other variants map to regions involved in immune regulation and inflammatory signaling, reflecting the polygenic nature of rheumatic disease risk. When these inherited susceptibilities coexist with benzene exposure, the combined effect may not operate through a single mechanism but rather create a biological context in which environmentally induced oxidative and metabolic perturbations exert stronger or more persistent consequences. Although our analyses did not identify statistically significant multiplicative or additive interactions, individuals with both high polygenic risk and elevated benzene exposure consistently showed the highest observed risks. Therefore, this pattern should be interpreted as a joint association rather than evidence of a gene–environment interaction.

This study has several notable strengths. First, the large sample size and prospective cohort design provided robust statistical power and reduced the potential for reverse causation. Second, the simultaneous evaluation of SLE, RA, and gout within a unified analytic framework enabled the identification of both shared and disease-specific associations. Third, the consistent findings across multiple sensitivity analyses reinforce the robustness of the results.

Several limitations merit consideration. First, ambient benzene exposure was estimated using residential address–based modeling, which may not fully capture individual-level exposure from occupational and indoor sources or during commuting. Residential mobility may also introduce exposure misclassification if changes in residence were not fully captured. Such exposure misclassification may have influenced the observed associations. Second, the UK Biobank population is predominantly of European ancestry, which may limit the generalizability of our findings to other ethnic populations. In addition, the PRSs used in this study were primarily derived and validated in populations of European ancestry and may have reduced predictive performance in other ancestry groups. Therefore, external validation in more ethnically and geographically diverse populations is warranted. Third, case ascertainment was based on linked hospital admission records, which may not capture milder or outpatient cases and could lead to underestimation of incidence or misclassification of disease onset. Fourth, benzene exposure may be correlated with other traffic-related air pollutants, such as PM_2.5_, NO_2_, and other volatile organic compounds. Therefore, residual confounding by co-exposure to these pollutants cannot be excluded, and the observed associations should not be interpreted as benzene-specific effects. Fifth, the relatively small number of incident SLE cases, particularly after stratification by PRS and benzene exposure, may have limited statistical power and resulted in imprecise estimates for some subgroup analyses. Finally, as in all observational studies, residual confounding cannot be ruled out despite careful covariate adjustment and the use of E-values. These limitations should be considered when interpreting the observed associations.

## 5 Conclusion

In summary, we investigated the associations of ambient benzene exposure and genetic susceptibility with the risk of incident RDs in the UK. Our study showed increased RA, SLE and gout risk among individuals with higher genetic susceptibility and elevated ambient benzene exposure. These findings highlight an association between ambient benzene exposure and RDs risk and underscore the importance of jointly considering environmental and genetic factors in future research.

### 5.1 Public health implications

Our findings suggest that long-term exposure to ambient benzene, even at relatively low environmental levels, is associated with higher risks of several rheumatic diseases. However, the observed increases in absolute disease occurrence were modest, and these findings do not establish that benzene exposure causes these diseases. For the public, the results support broader efforts to reduce unnecessary exposure to benzene and improve air quality, particularly because common sources of benzene include road traffic, fuel-related emissions, tobacco smoke, and industrial activities. Further studies are needed to determine whether reducing ambient benzene exposure can lower the risk of rheumatic diseases.

## Data Availability

Publicly available datasets were analyzed in this study. This data can be found here: Access to the UK Biobank data can be requested by means of a standard protocol (https://www.ukbiobank.ac.uk/register-apply/). This project was approved under application no. 641439.
