# Supplementary material for: Exposure to ambient benzene, genetic susceptibility, and incident rheumatic diseases in the UK
Source: Front Public Health. 2026 Oct 8;14:1931281. doi: 10.3389/fpubh.2026.1931281 (PMC13687248; doi:10.3389/fpubh.2026.1931281)
Supplement: Supplementary file 1 [file Data_Sheet_1.docx]

**Supplementary Files**

**Exposure to ambient benzene, Genetic Susceptibility, and incident rheumatic diseases in the UK**

**Table S1.** Disease definition based on ICD 10 code.

**Table S2**. The additive interactions between genetic susceptibility and ambient benzene exposure on the risk of incident SLE, RA and gout among UK participants.

**Table S3.** Associations of ambient benzene exposure with the risk of incident SLE, RA and gout among participants with length of living time at current address of more than 5 years.

**Table S4.** Associations of ambient benzene exposure with the risk of incident SLE, RA and gout among participants without poor self-reported health at baseline.

**Table S5.** Associations of ambient benzene exposure with the risk of incident SLE, RA and gout among white population.

**Table S6.** SNPs used for gout PRS construction.

**Figure S1.** Inclusion and exclusion criteria for the study population**.**

**Figure S2.** The distribution of ambient benzene concentration (μg/m^3^) between 2003 and 2010 in UK.

**Table S1.** Disease definition based on ICD 10 code.

| **ICD 10 codes** | **Diseases** |
| --- | --- |
| M320; M321; M328; M329 | Systemic lupus erythematosus, SLE |
| M050; M051; M052; M053; M058; M059; M060; M061; M062; M063; M064; M068; M069; M05; M06 | Rheumatoid Arthritis, RA |
| M100,M1000, M1001, M1002, M1003, M1004, M1006, M1007, M1008, M1009, M101, M102,M1020, M1024,M1025, M1027, M1029,M103, M1030,'M1036,M1037, M1039, M104, M1040, M1045,M1046,M1049, M109, M1090, M1091, M1092, M1093, M1094, M1095, M1096, M1097, M1098, M1099 | Gout |

**Table S2.** The additive interactions between genetic susceptibility and ambient benzene exposure on the risk of incident SLE, RA and gout among UK participants.

| **Outcome** | **Additive interaction** | | |
| --- | --- | --- | --- |
|  | RERI (95% CI) | AP (95% CI) | SI (95% CI) |
| Systemic lupus erythematosus, SLE |  |  |  |
| Intermediate risk | 0.18 (-0.56, 0.91) | 0.10 (-0.31, 0.51) | 1.29 (0.38, 4.36) |
| High genetic risk | 0.17 (-0.90, 1.24) | 0.04 (-0.21, 0.29) | 1.06 (0.74, 1.50) |
| Rheumatoid Arthritis, RA |  |  |  |
| Intermediate risk | 0.05 (-0.09, 0.20) | 0.04 (-0.07, 0.14) | 1.14 (0.77, 1.69) |
| High genetic risk | -0.01 (-0.17, 0.16) | -0.00 (-0.09, 0.08) | 0.99 (0.85, 1.17) |
| Gout |  |  |  |
| Intermediate risk | 0.08 (-0.08, 0.23) | 0.04 (-0.04, 0.12) | 1.08 (0.91, 1.29) |
| High genetic risk | 0.14 (-0.03, 0.32) | 0.05 (-0.01, 0.12) | 1.09 (0.98, 1.22) |

*Note.* Model adjusted for sex, age, household income, education, body mass index (BMI), drinking status, smoking status, outdoor time, and diet score. RERI: Relative Excess Risk due to Interaction; AP: Attributable Proportion due to Interaction; SI: Synergy Index.

**Table S3.** Associations of ambient benzene exposure with the risk of incident SLE, RA and gout among participants with length of living time at current address of more than 5 years.

| **Benzene exposure (μg/m^3^)** | **HR (95% CI)** | ***P* value** | ***P* for trend** |
| --- | --- | --- | --- |
| Systemic lupus erythematosus, SLE |  |  |  |
| Q1, lowest | 1 [reference] | NA | 0.017 |
| Q2 | 1.22 (0.91–1.62) | 0.189 |  |
| Q3 | 1.32 (0.98–1.76) | 0.066 |  |
| Q4, highest | 1.42 (1.06–1.91) | 0.018 |  |
| Per SD increases | 1.13 (1.04–1.24) | < 0.01 | NA |
| Rheumatoid Arthritis, RA |  |  |  |
| Q1, lowest | 1 [reference] | NA | < 0.001 |
| Q2 | 1.21 (1.13–1.30) | < 0.001 |  |
| Q3 | 1.18 (1.10–1.27) | < 0.001 |  |
| Q4, highest | 1.18 (1.10–1.27) | < 0.001 |  |
| Per SD increases | 1.05 (1.02–1.07) | < 0.001 | NA |
| Gout |  |  |  |
| Q1, lowest | 1 [reference] | NA | < 0.001 |
| Q2 | 1.12 (1.06–1.19) | < 0.001 |  |
| Q3 | 1.28 (1.21–1.36) | < 0.001 |  |
| Q4, highest | 1.26 (1.18–1.34) | < 0.001 |  |
| Per SD increases | 1.10 (1.08–1.12) | < 0.001 | NA |

*Note.* Model adjusted for sex, age, household income, education, body mass index (BMI), drinking status, smoking status, outdoor time, and diet score.

**Table S4.** Associations of ambient benzene exposure with the risk of incident SLE, RA and gout among participants without poor self-reported health at baseline.

| **Benzene exposure (μg/m^3^)** | **HR (95% CI)** | ***P* value** | ***P* for trend** |
| --- | --- | --- | --- |
| Systemic lupus erythematosus, SLE |  |  |  |
| Q1, lowest | 1 [reference] | NA | 0.055 |
| Q2 | 1.39 (1.03–1.88) | 0.030 |  |
| Q3 | 1.38 (1.01–1.88) | 0.042 |  |
| Q4, highest | 1.39 (1.02–1.91) | 0.039 |  |
| Per SD increases | 1.09 (0.99–1.20) | 0.065 | NA |
| Rheumatoid Arthritis, RA |  |  |  |
| Q1, lowest | 1 [reference] | NA | 0.002 |
| Q2 | 1.21 (1.13–1.29) | < 0.01 |  |
| Q3 | 1.17 (1.09–1.26) | < 0.01 |  |
| Q4, highest | 1.14 (1.06–1.23) | < 0.01 |  |
| Per SD increases | 1.04 (1.01–1.06) | < 0.01 | NA |
| Gout |  |  |  |
| Q1, lowest | 1 [reference] | NA | < 0.001 |
| Q2 | 1.15 (1.08–1.22) | < 0.01 |  |
| Q3 | 1.29 (1.22–1.38) | < 0.01 |  |
| Q4, highest | 1.23 (1.15–1.31) | < 0.01 |  |
| Per SD increases | 1.09 (1.06–1.11) | < 0.001 | NA |

*Note.* Model adjusted for sex, age, household income, education, body mass index (BMI), drinking status, smoking status, outdoor time, and diet score.

**Table S5.** Associations of ambient benzene exposure with the risk of incident SLE, RA and gout among white population.

| **Benzene exposure (μg/m^3^)** | **HR (95% CI)** | ***P* value** | ***P* for trend** |
| --- | --- | --- | --- |
| Systemic lupus erythematosus, SLE |  |  |  |
| Q1, lowest | 1 [reference] | NA | 0.024 |
| Q2 | 1.22 (0.92–1.62) | 0.160 |  |
| Q3 | 1.36 (1.03–1.81) | 0.032 |  |
| Q4, highest | 1.37 (1.02–1.84) | 0.034 |  |
| Per SD increases | 1.10 (1.01–1.21) | 0.030 | NA |
| Rheumatoid Arthritis, RA |  |  |  |
| Q1, lowest | 1 [reference] | NA | < 0.001 |
| Q2 | 1.19 (1.11–1.27) | < 0.001 |  |
| Q3 | 1.18 (1.10–1.27) | < 0.001 |  |
| Q4, highest | 1.17 (1.08–1.25) | < 0.001 |  |
| Per SD increases | 1.05 (1.03–1.08) | < 0.001 | NA |
| Gout |  |  |  |
| Q1, lowest | 1 [reference] | NA | < 0.001 |
| Q2 | 1.15 (1.08–1.21) | < 0.001 |  |
| Q3 | 1.29 (1.21–1.37) | < 0.001 |  |
| Q4, highest | 1.27 (1.19–1.35) | < 0.001 |  |
| Per SD increases | 1.10 (1.08–1.13) | < 0.001 | NA |

*Note.* Model adjusted for sex, age, household income, education, body mass index (BMI), drinking status, smoking status, outdoor time, and diet score.

**Table S6.** SNPs used for gout PRS construction.

| SNP | Effect allele | Beta |
| --- | --- | --- |
| rs75460349 | C | 0.203826 |
| rs6676150 | C | 0.113354 |
| rs1260326 | C | -0.12935 |
| rs73179273 | C | 0.113289 |
| rs34004016 | C | -0.128775 |
| rs4697701 | A | -0.340252 |
| rs2231142 | T | 0.497685 |
| rs79659488 | T | 0.193155 |
| rs62310002 | G | 0.342424 |
| rs114712651 | T | 0.313893 |
| rs1285875 | C | -0.0865073 |
| rs1165152 | G | 0.102879 |
| rs58233715 | G | 0.119602 |
| rs1171617 | T | 0.1096 |
| rs11227299 | G | 0.0994067 |
| rs3741414 | T | -0.0885698 |
| rs7212526 | T | -0.10456 |
| rs182368979 | T | 0.306305 |
| rs4645896 | A | 0.320543 |
| rs1064257 | G | 0.730664 |
| rs112395288 | T | 0.517293 |
| rs738409 | G | -0.11249 |

**Figure S1.** Inclusion and exclusion criteria for the study population.

**Figure S2.** The distribution of ambient benzene concentration (μg/m^3^) between 2003 and 2010 in UK.
